# Activated CD8^+^ T Lymphocytes Inhibit Neural Stem/Progenitor Cell Proliferation: Role of Interferon-Gamma

**DOI:** 10.1371/journal.pone.0105219

**Published:** 2014-08-18

**Authors:** Shuxian Hu, Jessica H. Rotschafer, James R. Lokensgard, Maxim C-J. Cheeran

**Affiliations:** 1 Center for Infectious Diseases and Microbiology Translational Research, Department of Medicine, University of Minnesota Medical School, Minneapolis, Minnesota, United States of America; 2 Department of Veterinary Population Medicine, College of Veterinary Medicine, St. Paul, Minnesota, United States of America; Temple University School of Medicine, United States of America

## Abstract

The ability of neural stem/progenitor cells (NSCs) to self-renew, migrate to damaged sites, and differentiate into neurons has renewed interest in using them in therapies for neurodegenerative disorders. Neurological diseases, including viral infections of the brain, are often accompanied by chronic inflammation, whose impact on NSC function remains unexplored. We have previously shown that chronic neuroinflammation, a hallmark of experimental herpes simplex encephalitis (HSE) in mice, is dominated by brain-infiltrating activated CD8 T-cells. In the present study, activated CD8 lymphocytes were found to suppress NSC proliferation profoundly. Luciferase positive (luc^+^) NSCs co-cultured with activated, MHC-matched, CD8^+^ lymphocytes (luc^−^) showed two- to five-fold lower luminescence than co-cultures with un-stimulated lymphocytes. On the other hand, similarly activated CD4^+^ lymphocytes did not suppress NSC growth. This differential lymphocyte effect on proliferation was confirmed by decreased BrdU uptake by NSC cultured with activated CD8 T-cells. Interestingly, neutralizing antibodies to interferon-gamma (IFN-γ) reversed the impact of CD8 lymphocytes on NSCs. Antibodies specific to the IFN-γ receptor-1 subunit complex abrogated the inhibitory effects of both CD8 lymphocytes and IFN-γ, indicating that the inhibitory effect of these cells was mediated by IFN-γ in a receptor-specific manner. In addition, activated CD8 lymphocytes decreased levels of nestin and Sox2 expression in NSCs while increasing GFAP expression, suggesting possible induction of an altered differentiation state. Furthermore, NSCs obtained from IFN-γ receptor-1 knock-out embryos were refractory to the inhibitory effects of activated CD8^+^ T lymphocytes on cell proliferation and Sox2 expression. Taken together, the studies presented here demonstrate a role for activated CD8 T-cells in regulating NSC function mediated through the production of IFN-γ. This cytokine may influence neuro-restorative processes and ultimately contribute to the long-term sequelae commonly seen following herpes encephalitis.

## Introduction

Over the past decade it has become well established that adult mammalian brains possess an inherent ability to regenerate [1]. This property of neurogenesis is realized by a small population of undifferentiated, self-renewing cells called neural stem/progenitor cells (NSC). The neurogenic cell population is housed predominantly in two well-described, discrete germinal centers (niches) within the brain, the subventricular zone (SVZ) of the lateral ventricles and the subgranular zone (SGZ) of the hippocampus. The neurogenic niche consists of a diverse population of cell types [2], which when examined at the population level exhibit distinct morphological and functional properties while still retaining their ability to proliferate and for multipotent differentiation [3], [4]. Astroglial-like Type 1 cells (also called B cells) are the top-level stem/precursor population, which in addition to symmetric division are able to divide asymmetrically to generate Type 2 progenitor cells. These intermediate Type 2 or C cells retain expression of stem/progenitor markers (like Sox2) and begin to express lineage specific markers, like doublecortin (DCX) [4] before they become A cells or neuroblasts.

Although several models have been proposed to delineate lineage relationships of different neural stem/progenitor cell types [5], it is very evident that the germinal niches provide complex and dynamic microenvironments necessary to sustain their self-renewal and multipotent phenotypes [6] and are influenced by both intrinsic and extrinsic signals that modulate neurogenic processes in the brain (reviewed in [7]). The inflamed brain microenvironment consequent to infection [8], ischemic stroke [9], or in neurodegenerative disease states, like Huntington's and Parkinson's disease [10], affects the composition and architecture of the SVZ resulting in changes in proliferation, migration and differentiation of NSCs (reviewed in [11]). Neurological injury, associated with concomitant acute and/or chronic inflammation, alters neurogenesis within these NSC niches. Hence, understanding the role and influence of inflammation on these germinal centers in the brain is essential to garner the potential for NSCs to regenerate/repair damaged brains [12], [13], [14].

T-cells have been postulated to play a major role in neurogenesis. Activated T-cells influence the recovery process regardless of the type of brain injury [15], [16]. In fact, it has been postulated that CD4^+^ T-cells may have a physiological role in maintenance of memory and learning. Impairment in CD4^+^ T-cells, either by genetic manipulation or immunological depletion, results in decreased hippocampal neurogenesis and significant impairment in performance on memory tests [17], [18], [19], [20], suggesting that peripheral immune cells and mediators may play a role in maintaining cognitive function under physiological conditions [16].

Contrary to their physiological role in promoting neurogenesis, T lymphocytes in pathological conditions are known to inhibit neurogenesis and altering T-cell function is often associated with favorable outcomes [21]. Immigrating myelin-specific T-cells during experimental autoimmune encephalitis (EAE) have been associated with decreased NSC proliferation. The chronic nature of the inflammation in EAE alters both the composition and the architecture of the SVZ, the NSC niche [22]. Similarly, larger infarct volumes resulting from ischemic brain injury have been linked to presence of activated T-cells in the brain [23], [24], [25], presumably driven by the neurotoxic effects of immune mediators generated by Th1 and Th2 lymphocytes [26]. Interestingly, T-cell deficiency also increased neural stem/progenitor proliferation during the acute stages of stroke [27]. This dichotomous function of activated T-cells may be related to its cytokine profiles generated during the inflammatory process following brain injury [28] and suggests that immune cells play an critical role in both the initiation and progression of neurogenesis.

Inflammation is an essential component of the host response that protects against brain infections. Our laboratory has demonstrated that the inflammatory response to HSV-1 infection clears virus from the brain, but leaves in its wake a chronic activated T [29], [30] and B lymphocyte [31] response, potentially serving critical surveillance functions. Persistent cytotoxic CD8 T-cells produce IFN-γ, a cytokine that both our laboratory and others have shown to alter NSC functions [32]. While a substantial number of studies have investigated the role of the inflammatory response in defense of and damage to the brain, little if anything is known about the impact of neuroimmune responses on NSCs in the context of viral encephalitis. In the present study, we utilized a co-culture system to determine the impact of activated T-cells on NSC proliferation and began to decipher the molecular cues associated with their effects on NSCs.

## Materials and Methods

### Ethics Statement

All experiments in this study using animals were conducted under protocols approved by the University of Minnesota Institutional Animal Care and Use Committee and in concordance with the Guide for the Care and Use of Laboratory Animals.

#### Neural stem/progenitor cell culture

Luciferase-expressing transgenic Balb/c mice were generated by backcrossing FVB mice expressing luciferase under the control of a β-actin promoter (Xenogen, Alameda, CA) into the Balb/c background for ten generations [31], [33]. Murine NSCs were cultured from the brains of E14.5 luciferase transgenic mice, Balb/c mice, or IFN-γR1 KO mice (CByJ.129S7(B6)-*Ifngr1^tm1Agt^*/J; Jackson Laboratory, Bar Harbor, Maine) as previously described [34], [35] with few minor modifications. Briefly, timed-pregnant mouse embryos (E14.5) were dissected in Hank's buffer (Sigma, St. Louis, MO). After decapitation and removal of skin, skull, and meninges, cerebral cortices were mechanically triturated in Hank's buffer and dissociated cells were collected and resuspended in serum-free NSC medium (DMEM/F12, 8 mM glucose, glutamine, 20 mM sodium bicarbonate and N2 Plus supplement, R&D Systems, Minneapolis, MN). Cells (2 × 10^5^) were then plated on 10-cm diameter dishes, pre-coated with 15 µg/ml poly-L-ornithine and 1 µg/ml bovine fibronectin, and cultured in NSC medium supplemented with bFGF (20 ng/ml; R&D Systems) and EGF (20 ng/ml; R&D Systems). NSC monolayer cultures, maintained at 37°C (5% CO_2_) in NSC growth medium (with bFGF and EGF supplemented daily and half-media changes every 48 h) were sub-cultured when 60–70% confluence was attained and re-plated at a density of 2×10^5^ cells per 10 cm dish or 2×10^3^ cells per well of 24-well plates. This culture was considered as passage 1. NSC cultures between passages 1–3 were used throughout this study. Near homogeneity (>98% positive) in Sox2 and Nestin (stem cell markers) expression was observed in these cultures, with a proportion (41.2 ± 5.1%) of the cells expressing doublecortin (neuroblast marker) in addition to Sox2. GFAP expression was not detected in cultured NSCs.

### Lymphocyte isolation and stimulation

Spleens and lymph nodes were collected from naïve Balb/C mice, raised under SPF conditions, as previously described [29]. Briefly, single cell suspensions of splenocytes were depleted of red blood cells by treatment with 0.87% ammonium chloride, washed twice, and cell viability was confirmed using trypan blue. T lymphocyte subsets were isolated from RBC free, lymph node cells and splenocytes using a ferrofluid based system (R&D Systems Inc., Minneapolis, MN), according to the manufacturer's instructions. Untouched CD4 and CD8 lymphocytes were enriched from lymph node and spleen using a negative selection based antibody cocktail that enriches for each lymphocyte population. Enrichment of lymphocyte subtypes were >90% in all experiments.

Enriched CD4 and CD8 T-cells were co-cultured with Luc^+^ NSCs 24 h after seeding the stem cells. Cell ratios of NSC and lymphocytes were calculated based on number of NSC seeded in each well. All experiments were conducted using passage 1 (P1) -P3 NSC cultures, which were determined to have similar doubling rates. Lymphocytes were stimulated in co-cultures using a mixture of monoclonal antibodies to CD3 (5 µg/ml; clone 17A2) and CD28 (5 µg/ml; clone 37.5; BioXcell, West Lebanon, NH). Appropriate controls with and without activating antibodies or lymphocytes were used to compare NSC proliferation in the presence of lymphocytes. Lymphocyte conditioned medium was prepared from cell free supernatants of stimulated or unstimulated lymphocytes cultured in NSC growth medium for 72 h. Half of the NSC growth medium was replaced with lymphocyte conditioned medium (supplemented with EGF & FGF at the time of co-culture) to investigated the role of soluble factors in lymphocyte-mediated effects on NSCs. No additional NSC-media changes were done in all cultures tested for the 72 h duration of the experiment.

### Luciferase assay

Luciferase activity levels from luciferase (luc)^+^ murine NSCs were used as an indicator of cell proliferation. At 24–72 h post treatment, cell lysates were prepared using the lysis reagent per manufacturer's directions (Promega, Madison, WI). Luciferase assays were performed using 10 µl of cell lysate and 10 µl of the luciferase substrate provided in an opaque 96-well plate. Luminesce levels was measured on a luminometer (Luminoskan, Thermo Fisher Scientific, Walthan MA) using a 10 s read time. NSC growth characteristics that were measured by luciferase activity was repeated in similarly designed, selected experiments using ^3^[H]-thymidine incorporation assay, as previously described [29].

### NSC Proliferation Assay

E14.5 embryonic NSCs were labeled with 5 nM fixable proliferation dye eFluor450 (eBioscience, San Diego, CA) for 5 minutes at 37°C and quenched with 5 mL one to one ratio of complete NSC media (DMEM/F12 with N2 supplement) and 7.5% bovine serum albumin. About 15×10^5^ labeled cells were cultured in NSC media or in the presence of 20 pg/mL interferon-gamma (IFN-γ), 5×10^5^ CD8 T-cells either left unstimulated, or stimulated overnight (with 10 µg/mL each of anti-CD3 and anti-CD28) prior to co-culture for 72 hours. Cells were immunostained for CD45 (PerCP-Cy5.5; eBioscience) and Sox2 (eFluor670; eBioscience), and assayed on a BD FACSCanto for cell numbers with AccuCount blank particles for absolute quantification. Quantification of cell proliferation was performed by measuring dye dilution in the eFluor450 labeled cells, per the manufacturer's instructions. Cell proliferation analysis was done using FlowJo software (Treestar Inc. Ashland OR)

### Analysis of NSC by Flow Cytometry

NSCs were stained for nestin expression by fixing cells in 4% paraformaldehyde (in PBS) for 20 min and incubating in SAP buffer (2% FCS, 0.5% saponin, and 0.1% sodium azide in PBS) containing anti-rat nestin-PE monoclonal antibody or IgG-PE isotype antibody (R&D Systems, MN). Following incubation for 30 min at room temperature the cells were washed once with SAP buffer, once with PBS, resuspended in 400 µl of PBS and analyzed on a FACS Canto flow cytometer (Becton-Dickinson, Mountain View, CA). At least ten thousand events were analyzed using FlowJo software (Tree Star Inc. Ashland OR). Doublets were eliminated from the analysis of live cell gated events using data obtained on their scatter height and width geometry. Only singlet cells were used in flow cytometry analysis.

NSCs were immunostained by fixing and permeabilizing cells (BD Cytofix/Cytoperm kit (Becton-Dickinson) prior to staining with anti-Human/Mouse Sox2-eFluor 660 (Clone-Btjce; eBioscience, San Diego, CA) and rabbit anti-mouse Ki-67 (Abcam, Cambridge, MA). To visualize Ki67 staining, a PE labeled goat anti-rabbit IgG (Becton-Dickinson) secondary antibody was used. Antibodies were incubated at 4°C and 50 µL blank AccuCount particles (Spherotech, Lake Forest, IL) were added immediately prior to data acquisition for cell quantification, per manufacturer's directions. A minimum of 30,000 events was collected on a FACS Canto flow cytometer (BD, Mountain View, CA).

### BrdU incorporation assay

NSC and T lymphocyte cultures were pulsed with 10 µM BrdU 72 h after co-cultures were instituted. Subsequent to a 3 h BrdU pulse, cells were dissociated from the culture plate using Ca^++^/Mg^++^ free Hanks blocked with 1 µg Fc Block (BD Bioscience, San Jose, CA). The cell suspension was then immunostained for CD45 using an APC conjugated antibody (BD Biosciences, San Jose, CA) in FACs buffer containing 2% mouse serum and 2% rat serum. BrdU positive cells were stained using a FITC BrdU flow staining kit (BD Biosciences, San Jose, CA) per manufacturer's instructions. Briefly, cells were permeabilized in Cytofix/Cytoperm buffer and incubated with BD Cytoperm Plus Buffer open the nuclear membrane. The cells were then incubated with 30 µg DNase at 37°C for 1 h and stained with FITC-conjugated anti-BrdU antibody (BD Biosciences, San Jose, CA). The samples were read on a BD FACS Canto and BrdU incorporation was analyzed using the FlowJo software (Tree Star Inc. Ashland OR). Live cell gates were applied to identify intact cells and CD45+ cells were gated out to remove lymphocytes from NSC analysis.

### Cytotoxicity analysis in NSC-lymphocyte co-cultures

To identify NSC apoptosis in co-cultured cells, they were removed from the plate gently using Ca++/Mg++ free Hank's balanced solution for dissociation. Lymphocytes were identified by staining with APC-conjugated anti-CD45 antibody (BD Biosciences) in FACs blocking buffer (1 µg Fc block, 2% normal mouse and rat serum). The cell suspension was washed twice with cold PBS and re-suspended in 100 µL of Annexin V binding buffer with FITC-conjugated Annexin V (Invitrogen, Carlsbad, CA). Cells were then stained with propidium idodide to identify dead cells and read on a FACS Canto within 1 hour of the assay. Data were analyzed using FlowJo software.

### IFN-γ ELISA

Cell free supernatants from treated and untreated NSC and lymphocyte cultures, collected at 72 h post treatment, were used to measure IFN-γ production using an ELISA kit (R&D Systems) according to the manufacturers' protocol.

### Immunocytochemistry for stem cell differentiation

Murine NSCs were cultured in 24-well tissue culture plates alone, with unstimulated CD8 T lymphocytes, or with pan-stimulated CD8 T lymphocytes as described in the Lymphocyte Isolation and Stimulation methods section. Cells were cultured for 72 h, the maximum duration of the experiment, without additional media change during this period. NSCs were fixed with 2% paraformaldehyde and permeabilized in PBS with 0.3% Triton-X 100 (Sigma-Aldrich, St. Louis, MO). Fixed cells were then treated with PBS containing 5% bovine serum albumin and 5% goat serum to block non-specific antibody binding. Cells were incubated with primary antibodies against GFAP (Dako Laboratories, Carpinteria, CA), β-III tubulin (Tuj-1; R&D Systems, Minneapolis, MN), or O4 (R&D Systems) followed by secondary antibodies: goat anti-rabbit AlexaFluor 488 for GFAP, and biotinylated anti-mouse Ig for β-III tubulin and O4 (Life Technologies, Grand Island, NY). DAPI was used as a counterstain. Cells were imaged on an Olympus IX70 inverted fluorescence microscope.

## Results

### Activated CD8 T-cells inhibit neural stem cell proliferation

**Figure 1:**
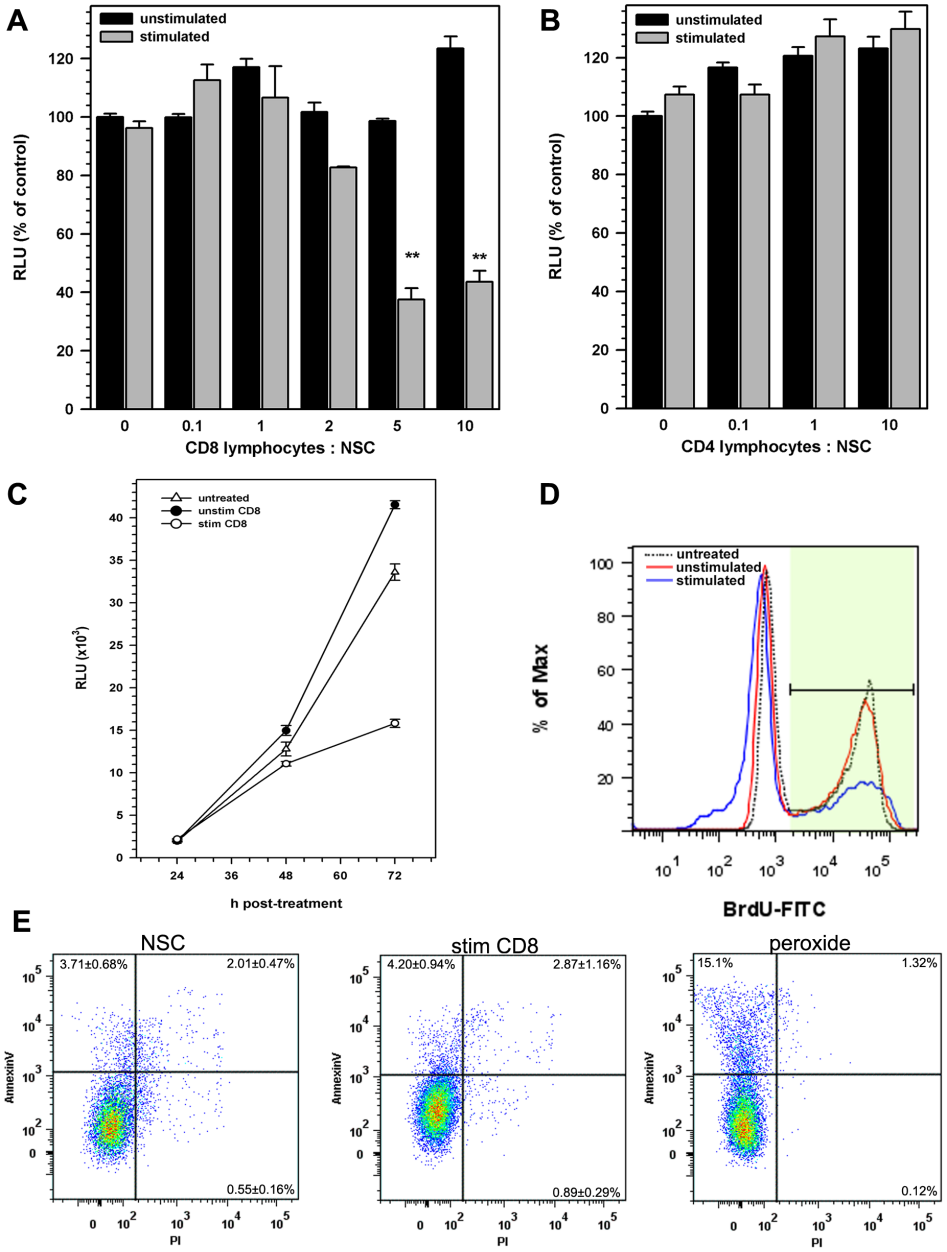
CD8^+^ lymphocytes inhibit NSC proliferation. Luc^+^ NSCs obtained from E14.5 d luciferase transgenic mice were co-cultured with **(A)** CD8+ or **(B)** CD4+ lymphocytes isolated from MHC-matched (Luc^-^) Balb/C mice 24 h after seeding Luc^+^ NSCs. Lymphocytes in the co-cultures were either left unstimulated (black bars) or were stimulated with a mixture of anti-CD3 and anti-CD28 antibodies (5 µg/ml). After 72 h of stimulation, culture lysates were analyzed for luciferase activity. Data presented are pooled from 2–5 separate experiments with each treatment performed in triplicate (mean ± SEM). **(C)** Kinetics of NSC growth *in vitro* measured as a function of NSC luciferase activity. Rate of NSC growth left untreated (open triangle) was compared to rate of growth in co-culture with unstimulated lymphocytes (closed circles) or activated CD8 lymphocytes (open circles). Data presented are pooled from two separate experiments with each treatment performed in triplicates (mean ± SEM). **(D)** NSC proliferation, measured by their ability to incorporate BrdU, was assessed by flow cytometry. Total live cells were gated for analysis and lymphocytes were excluded by gating out CD45^+^ cells. A representative histogram of two independent experiments showing BrdU uptake by CD45^−^ NSC co-cultured with activated CD8 lymphocytes (blue) and compared to those co-cultured with unstimulated (red) or untreated (dotted line) cells. **(E)** Representative dot plots showing Annexin-V binding and propidium iodide uptake by untreated NSCs (NSC), NSCs cultured with antibody-stimulated CD8 T lymphocytes (stim CD8), NSCs treated with hydrogen peroxide for 1 hour (peroxide). ** p<0.001 vs untreated NSC.

To determine if activated T lymphocytes altered NSC functions, stem cells isolated from the cortices of luciferase (luc+) transgenic mice embryos (E14.5) were co-cultured with stimulated or unstimulated major histocompatibility complex (MHC)-matched wild type (luc-) lymphocytes, thereby permitting analysis of NSC growth in culture by measuring luciferase activity. Initial experiments demonstrated that peak luciferase activity was observed 72-96 h post seeding. CD8 lymphocytes, activated by crosslinking CD3 and CD28 with monoclonal antibodies (Mab), inhibited NSC growth in a dose-dependent manner (Fig. 1A). Activated CD8 T-cells inhibited NSC growth at co-culture ratios as low as 2 lymphocytes per NSC (82.7% ± 0.4%). At higher ratios (5–20 lymphocytes per NSC), inhibition ranged from 22% to 64% of luminescence observed in similar untreated control cultures. Average luminescence in NSCs treated with 10 activated CD8 lymphocytes per NSC decreased to 43.6% ± 3.9% of that observed in untreated cultures. Similarly reconstituted co-cultures using unstimulated CD8 lymphocytes did not alter NSC growth (123.4% ± 1.2% with 10 unstimulated CD8 cells per NSC; Fig. 1A).

In contrast to CD8 lymphocytes, NSC growth was not inhibited by similarly activated CD4 lymphocytes using the same ratios (up to 20:1) of lymphocytes to NSC (Fig. 1B). The suppression of NSC growth by activated CD8 cells was greatest at 72 h post seeding NSC cultures (Fig. 1C), while co-culture with CD4 T-cells did not alter the kinetics of NSC growth in culture (data not shown). To further determine if the observed NSC growth inhibition was a consequence of suppressed cell proliferation, lymphocyte-treated and untreated cultures were evaluated for BrdU uptake at 72 h post constitution. Flow cytometry analysis for BrdU incorporation demonstrated that approximately 55% of NSCs in culture underwent active division at 72 h (Fig. 1D). While co-cultures with stimulated CD8+ T-cells suppressed BrdU uptake by NSCs (∼33.3%), unstimulated lymphocytes did not alter NSC proliferation (Fig. 1D). No change in NSC BrdU uptake was observed when treated with anti-CD3 or -CD28 antibodies alone or when cultured with activated or unstimulated CD4 lymphocyte (data not shown).

To exclude the possibility that CD8 T-cell inhibition of NSC growth was mediated by cytotoxicity, NSCs co-cultured with activated and unstimulated CD8 cells were assessed for annexin V staining, along with changes in cell permeability to propidium iodide (PI), to evaluate cell death. CD45 (+) staining was used to separate lymphocytes and NSC for flowcytometry analysis. No differences in annexin V binding or PI incorporation were observed in NSCs after 72 h co-culture (Fig. 1E). On average, AnnexinV staining was seen in 3–5% of NSC cultured with or without lymphocytes compared to 15% AnnexinV binding to NSC treated with hydrogen peroxide for 3 h prior to analysis (Fig. 1E).

### CD8 T-cells inhibit NSC proliferation through the production of Interferon gamma (IFN-γ)

**Figure 2:**
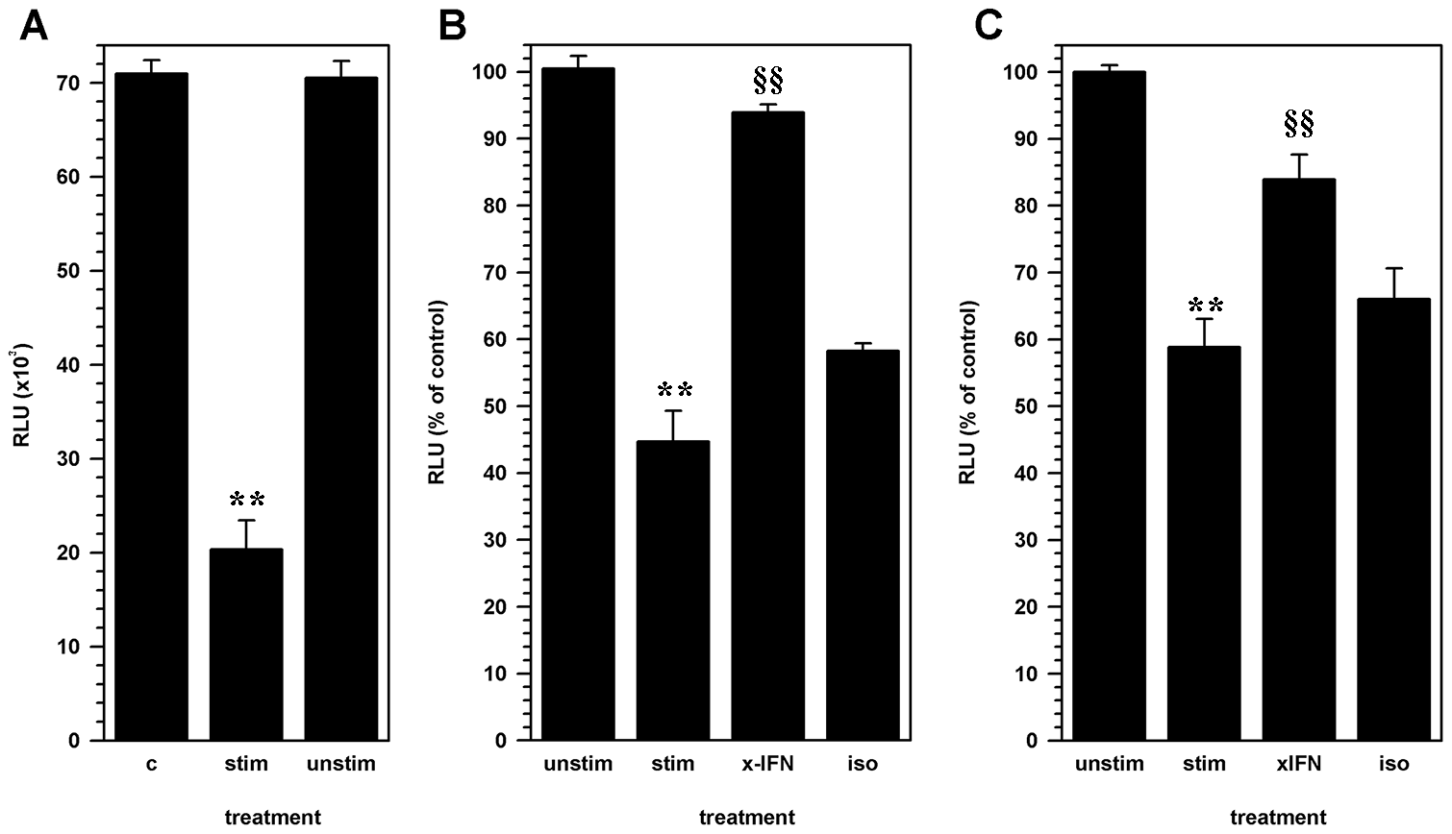
The effect of CD8 lymphocytes on NSC proliferation is mediated through IFN-γ. Luc^+^ NSC cultures were treated with cell-free supernatants from activated CD8 lymphocytes 24 h after seeding cultures. **(A)** Luciferase activity measured from untreated NSCs (C) was compared to cultures treated with conditioned medium from antibody-activated CD8 lymphocyte (stim) or unstimulated CD8 lymphocytes (unstim). Pooled data from two separate experiments are presented. **(B)** Neutralizing antibodies to IFN-γ (x-IFN; 1 mg/ml) or an isotype control antibody (iso) was added to the activated-lymphocyte conditioned media prior to treatment of NSCs. Relative luminescence from NSC cultures treated with conditioned medium from unstimulated lymphocytes (unstim) or activated CD8 cells (stim) and treated activated-lymphocyte conditioned media are presented as a percent of untreated cultures. Pooled data from 3 independent experiments are presented. **(C)** NSC-lymphocyte co-cultures constituted with antibody-stimulated CD8 T-cells (stim) were treated with either neutralizing anti-IFN-γ antibody (xIFN), or an isotype antibody (iso). Relative luminescence intensity (RLU) from treated co-cultures and co-cultures with unstimulated CD8 T-cells (unstim) are presented as a percentage of untreated control NSCs. Pooled data from 3 independent experiments are presented (mean ± SEM; ** p<0.001 vs. untreated NSC; §§ p<0.001 vs. stimulated CD8 T-cell-NSC co-culture).

Given that activated CD8 lymphocytes suppressed NSC proliferation without inducing cytotoxicity, the next experiments were designed to determine if soluble factors produced by activated T-cells suppressed NSC growth. CD8 T-cells were stimulated with the anti-CD3/CD28 antibody cocktail for 72 h to generate conditioned medium. Conditioned medium from stimulated and unstimulated CD8 lymphocytes was added to NSC cultures 24 h after seeding. Luminescence activity was significantly decreased in NSCs treated with conditioned medium from stimulated CD8 T-cells (24.7 ± 3.0 × 10^3^ RLU; p<0.001) reaching up to an average of 44.7% ± 4.36% of luminescence of untreated NSC (45.2 ± 2.8 ×10^3^ RLU). Conditioned medium from unstimulated CD8 lymphocytes did not alter NSC growth (100.5% ± 1.9%, Fig. 2A).

Previous studies from our laboratory have shown that IFN-γ, a T-cell cytokine, inhibits murine NSC proliferation [32]. To determine if IFN-γ was produced by CD8 T-cells when stimulated in co-culture with NSCs, cytokine levels were measured from culture supernatants by capture ELISA method. IFN-γ levels in the NSC-CD8 lymphocytes co-cultures (at 1:10 ratio) reached concentrations of 30.4 ± 3.9 pg/ml, 72 h after CD3-CD28 antibody stimulation. No detectable IFN-γ production was observed in untreated NSC cultures supernatants, in co-cultures with unstimulated CD8 T-cells or stimulated or unstimulated CD4 T cells (Table S1).

To determine if inhibition of NSC proliferation was mediated by IFN-γ, conditioned medium from stimulated CD8 cells was treated with neutralizing, anti-IFN-γ antibody. Treatment with IFN-γ neutralizing antibodies reversed the inhibitory effect of activated CD8 lymphocyte conditioned medium from 44.7% ± 4.36% to 93.9 ± 1.2% of control (p<0.001), while an isotype control antibody did not affect inhibition of NSC proliferation (Fig. 2B). The blocking effect of neutralizing antibodies to IFN-γ was also demonstrated using the co-culture system where activated CD8 T-cells remained in contact with NSC (Fig. 2C).

**Figure 3:**
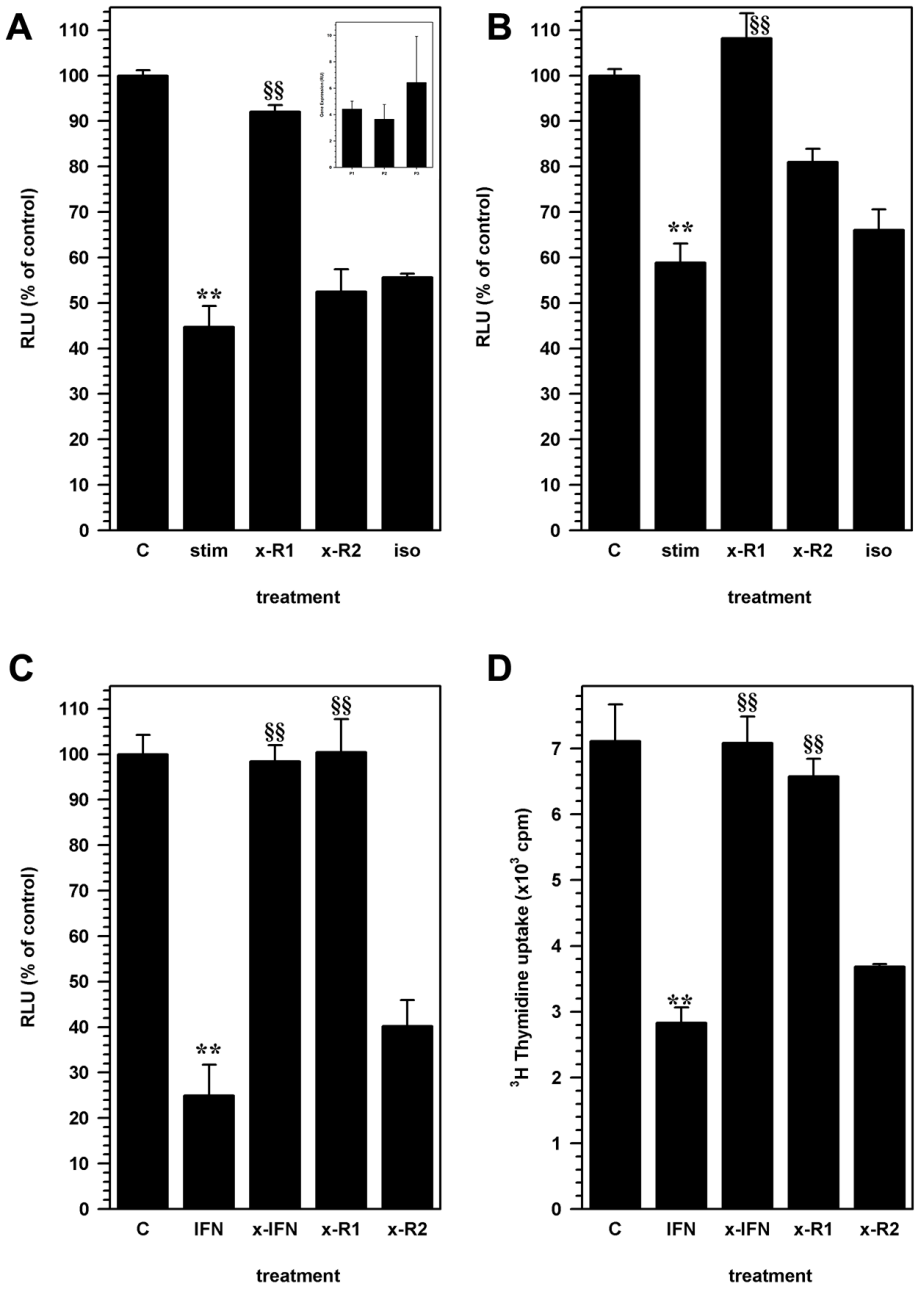
IFN-γ inhibition of NSC proliferation is mediated through the IFN-γ receptor. NSC cultures were treated with antibodies to IFN-γ alpha receptor (R1) subunit (x-R1) or IFN-γ beta receptor (R2) subunit (x-R2). **(A)** Relative luminescence units (RLU) from untreated control NSC cultures (C) were compared to cells treated with stimulated CD8 lymphocytes conditioned medium alone (stim), or cells treated with anti-IFN-γR1 (x-R1) or IFN-γ R2 (x-R2) or isotype control antibodies along with lymphocyte conditioned medium. (inset) Relative expression (RU) of IFN-γ R1 by Real-time RT-PCR analysis in NSC for obtained from first (P1), second (P2) and third (P3) passages in culture. Data are presented as average expression from 3 cultures (assayed in triplicate) at each passage (± SEM). **(B)** Co-cultures of NSCs with activated CD8 lymphocytes were either untreated (stim), or treated with anti-IFN-γR1 (x-R1), IFN-γ R2 (xR2) or isotype control antibodies (iso) to determine the role of lymphocyte-derived IFN-γ on stem cell proliferation. Luminescence intensity values are compared to similar untreated NSC cultures (C) and are presented as a percentage of untreated control cells. Data presented are pooled from 3 separate experiments (mean ± SEM). **(C)** The specificity of the IFN-γ mediated inhibition of NSC proliferation, mediated through the IFN-γ receptor, was recapitulated using recombinant murine IFN-γ (IFN) in the NSC culture medium. Anti-IFN-γ antibody (x-IFN), anti-IFN-γR1 antibody (x-R1) or anti-IFN-γR2 antibody was used to reverse the IFN-γ effect compared to untreated NSC cultures (C). Luminescence data are presented as a percentage of RLU from similar untreated NSC cultures. **(D)** Thymidine uptake assay was used to measure the effect of IFN-γ treatment (IFN) on NSC proliferation. IFN-γ effect on thymidine uptake was antagonized by treating with neutralizing antibodies to INF-γ (xIFN), IFN-γR1 (x-R1) but not by antibodies to IFN-γR2 (x-R2). Graphs represent pooled data obtained from 2–5 separate experiments with treatments performed in triplicates (mean ± SEM). ** p<0.001 vs. untreated NSC and §§ p<0.001 vs. stimulated CD8 T-cell or IFN-γ treated NSC.

The IFN-γ receptor is composed of two chains, the alpha (R1 subunit) and beta chains (R2 subunit; [36]). Previous studies have shown that murine NSCs express functional IFN-γ receptors [37]. We verified, using a quantitative RT-PCR, that IFN-γR1 was expressed in NSC from all passages of culture used in this study (Fig. 3A inset). NSC treatment with blocking antibodies to the IFN-γ R1 subunit significantly (p<0.001) reversed the inhibitory effects of activated CD8 lymphocyte supernatants (Fig. 3A). The inhibitory effect of CD8 cells was also observed in co-cultures of activated CD8 T-cells and NSC (Fig. 3B). However, inhibition of NSC proliferation was not affected by treatment with anti-IFN-γR2 (Fig. 3) antibody, indicating that IFN-γ binding to the alpha subunit induced the effects mediated by CD8 lymphocytes. Inhibition of proliferation was recapitulated in NSC cultures treated with recombinant murine IFN-γ (20 pg/ml) by measuring luciferase activity (Fig. 3C) and using a thymidine incorporation assay (Fig. 3D). Treatment with anti-IFN-γ and anti-IFN-γ R1 abrogated IFN-γ mediated effects, but inhibition was not affected by treatment with anti-IFN-γ R2 (Fig. 3C & D) or isotype control antibodies.

### Activated CD8 lymphocytes inhibit nestin and SOX2 expression in NSC

**Figure 4:**
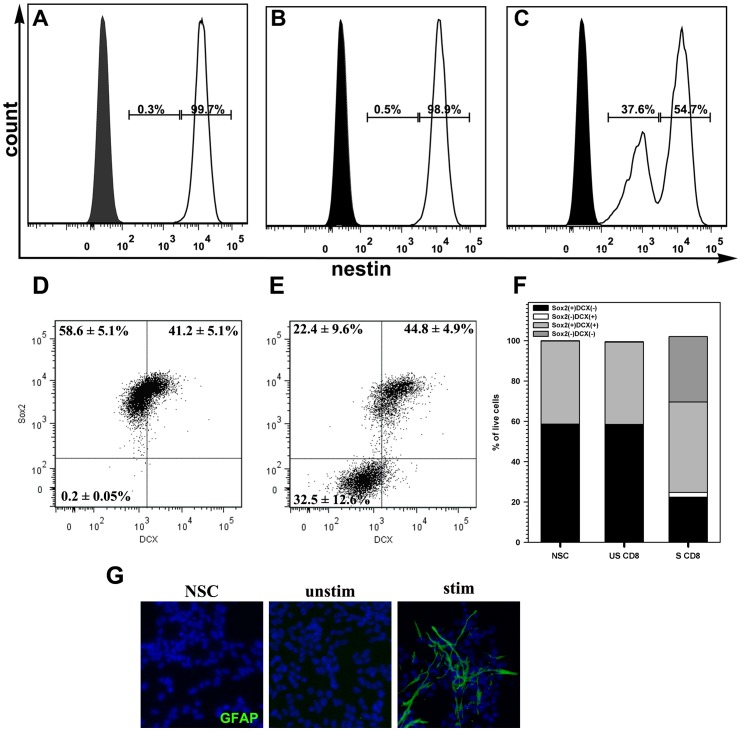
Activated CD8 lymphocytes initiate NSC differentiation. Co-cultures of NSC and lymphocytes were assessed for Nestin (a stem cell marker) expression by flowcytometry. NSCs were analyzed on a live cell gate after exclusion of CD45^+^ lymphocytes. **(A)** Histogram showing nestin immunoreactivity in untreated NSCs (open), compared to isotype antibody staining (shaded). Nestin expression in NSCs was measured 72 h after co-culture with **(B)** un-stimulated CD8 lymphocytes and **(C)** with activated CD8 lymphocytes. The amplitude of nestin expression and proportion of nestin-expressing cells decreased when NSC were co-cultured with activated CD8^+^ lymphocytes. Representative data from 2 separate experiments are presented. **(D)** Representative dot plots showing expression of Sox2 and doublecortin (DCX) on untreated NSCs or **(E)** NSCs cultured with antibody-stimulated CD8 T lymphocytes. **(F)** Stacked bars demonstrating changes in ratios of NSCs phenotypes, defined by Sox2 and doublecortin (DCX) expression, in untreated (NSC) cultures, NSCs cultured with unstimulated CD8 T-cell (US CD8), and NSCs cultured with antibody-stimulated CD8 T-cells (S CD8). **(G)** Immunohistochemistry of cultured NSCs showing differentiation into GFAP(+) cells when treated with stimulated CD8 T cell (stim). Untreated (NSC) NSCs and those co-cultured with unstimulated CD8 T cells show no GFAP immunostaining (green fluorescence). Nuclei were counterstained with DAPI (blue).

To determine if activated CD8 T-cells altered NSC phenotypes, expression of the stem cell and progenitor cell markers, nestin or Sox2 and doublecortin (DCX), were evaluated by flow cytometry. All NSC cultures used during these experiments had >98% of cells that expressed nestin and Sox2. Upon co-culture with activated CD8 lymphocytes, nestin expression on NSCs decreased in magnitude. Nestin expression was high in all untreated NSCs (Fig. 4A), and its expression was not altered following culture with unstimulated CD8 lymphocytes (Fig. 4B). In contrast, CD8 lymphocyte-treated NSCs expressed both high and intermediate levels of nestin (Fig. 4C). Although total number of NSCs expressing nestin did not alter significantly, approximately 38% of the cells showed lower fluorescence intensities of nestin immunoreactivity and ∼55% continued to express the protein at high levels.

Similarly, about 50% decrease in Sox2(+) expression was observed when NSCs were co-cultured with stimulated CD8 T-cells (Fig. 4D). NSC cultures that were left untreated or co-cultured with unstimulated CD8 T-cells showed two distinct CD45(−) cell populations when immunostained for both Sox2 and DCX; the Sox2(+)DCX(−) NSCs and Sox2(+)DCX(+) neuroblasts (Fig. 4E & F). CD45 immunostaining was used to exclude T-cells from the analysis. When co-cultured with activated CD8 T-cells, Sox2 expression decreased in the Sox2(+)DCX(−) population but the proportion of Sox2(+)DCX(+) cells remained unaltered, further indicating that CD8 T-cells may induce differentiation in a distinct subset of stem cells. To further validate that NSCs enter an altered differentiation state in response to activated CD8 T cells, co-cultures were stained for Tuj1, O4, and GFAP to assess NSC differentiation towards neurons, oligodendrocytes, and astrocyte-like cells, respectively. Untreated NSC cultures did not express GFAP or O4 but showed limited number of cells with Tuj-1 immunostaining (<1% of cells). However, a large proportion of cells expressed GFAP when co-cultured with stimulated CD8 T cells compared to untreated (NSC) cultures or co-cultures with unstimulated CD8 T cells (Fig. 4G). There was no observed differences in the limited Tuj-1 immunostaining in NSC cultures after 3 days in co-culture with activated CD8 T cells. O4 immunostaining was not detected in CD8 T-cell treated NSC cultures.

### Absence of IFN-γ R1 abrogates the inhibitory effects of activated CD8 T-cells on NSC proliferation

**Figure 5:**
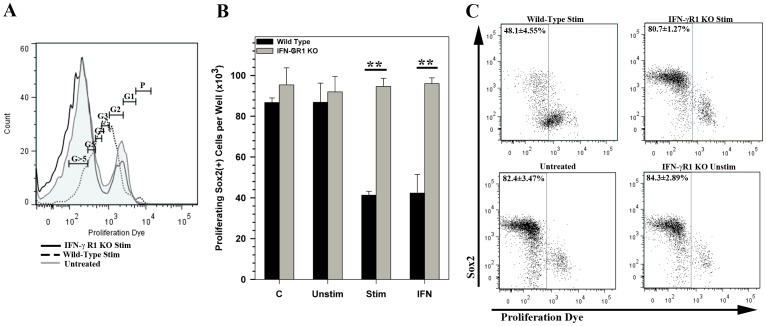
IFN-γR1 knockout NSCs are resistant to CD8 T-cell mediated inhibition of proliferation. NSCs were labeled with eFluor670 proliferation dye and were cultured alone (untreated), with 20 pg/mL IFN-γ (IFN) or in the presence of unstimulated (Unstim) or stimulated CD8 T cells (Stim). **(A)** Representative flow histograms of stimulated CD8 T cell treated and untreated NSCs demonstrating dilution of labeling dye fluorescence corresponding to cell divisions. Gates for generation of progeny was allocated based on progressive halving of the fluorescence measured in dividing NSCs (calculated using a cell proliferation algorithm in the FlowJo software). Proliferation of IFN-γR1 KO NSCs when co-cultured with stimulated CD8 T-cells (black line) compared to untreated NSCs (untreated, gray tinted line), or wild-type NSCs cultured with stimulated CD8 T-cells (dotted line) is shown. **(B)** Quantification of total number of proliferating Sox2(+) NSCs in shown under each culture condition for wild type and IFN-γR1 KO NSCs. Data are presented as an average of three replicates ± SEM. ** p<0.01. **(C)** Representative dot plots showing Sox2 immunolabeling vs dilution of proliferation dye fluorescence observed in wild type (upper left) and IFN-GR1 KO NSCs (upper right) co-cultured with stimulated CD8 T-cells. Untreated NSC cultures (lower left) and those co-cultured with unstimulated CD8 T-cells (lower right) show no difference in proliferation or Sox2 expression. Gates are drawn to indicate cells that have undergone five to seven rounds of division (or proliferation dye dilution) post culture and the percentages of proliferating cells are presented as average ± SEM.

To further confirm that activated CD8 T-cells mediated their effects on NSCs through an IFN-γ receptor mediated mechanism, NSC cultures derived from IFN-γ R1 KO mice were assessed for both proliferation and expression of Sox2 in the presence or absence of activated T cells. In a dye dilution proliferation assay, eFluor670 labeled NSCs showed a graded decrease in fluorescence intensity, with cells reaching up to >5 divisions in 72 h under proliferative culture conditions (Fig. 5A; untreated). The majority of wild-type NSCs co-cultured with stimulated CD8 T-cells showed an arrest in proliferation after 2 to 4 cell divisions. However, this arrest in proliferation was not observed in IFN-γ R1 KO NSCs, which proliferated in a manner similar to untreated cells (Fig. 5A). Wild-type and IFN-γ R1 KO cells that were untreated or treated with unstimulated CD8 T-cells showed similar patterns of proliferation, in that majority of the cells (∼80%) had undergone >5 cell divisions.

When co-labeled for Sox2 expression, we found that IFN-γ R1 KO Sox2(+) cells were unaffected by the inhibitory influence of activated CD8 T-cells on NSC proliferation (Fig. 5B). Interestingly, arrested proliferation occurred in cells with decreased Sox2 expression.

## Discussion

Data presented in this study demonstrate that activated CD8 T-cells inhibit NSC proliferation. Luciferase(+) NSCs co-cultured with activated CD8^+^ T-cells exhibited not only decreased total bioluminescence, indicative of lower cell numbers, but also showed decreased BrdU uptake by dividing cells. This decrease in cell number was not associated with an increase in cytotoxicity, a well-known CD-8 T-cell effector function. On the contrary, the inhibition of NSC proliferation was mediated via soluble factors produced by activated CD8 cells, effects of which could be abolished by neutralizing IFN-γ activity in the co-culture system. Interestingly, this inhibitory effect on NSCs was not observed in co-cultures with similarly activated CD4 T-cells. We found that antigen-independent activation of CD4 T-cells, using CD3 and CD28 antibodies, did not induce detectable levels of IFN-γ, explaining their lack of effect on NSCs. Previous studies have shown that cytokine production by CD4 T-cells is dependent on antigen driven mechanisms [38] and IFN-γ production is restricted to differentiated T helper-1 cells [39], [40]. Furthermore, co-culture with activated CD8 T-cells drives NSCs into a differentiation pathway, an effect that has been previously attributed to IFN-γ [41], [42].

Brain injury resulting from either infectious [29], [30] or non-infectious insults [24], [43], have been shown to result in CD8 T-cell recruitment to sites of brain damage. Our laboratory has previously demonstrated that in response to herpes viral infections, activated CD8 T-cells accumulate in the brain long after the virus is cleared [29], [30]. Retention and maintenance of activated CD8 T-cells in damaged CNS tissue is not unique to infectious etiology, but is observed in models of ischemic stroke [44] and aseptic cerebral injury [45]. Notably, this accumulation of CD8 lymphocytes occurs without apparent replenishment from peripheral lymphoid tissues and is mediated through both antigen-dependent and -independent mechanisms [46]. Several studies have demonstrated that CD8 lymphocytes contribute to tissue damage, largely mediated by their cytotoxic effector functions [24], [43]. We demonstrate that antigen-independent activation of CD8 T-cells robustly inhibits NSC proliferation, without associated cytotoxicity. Recently, we reported that endogenous NSC proliferation is markedly reduced during chronic herpes encephalitis [33]. This decrease in NSC proliferation coincides with the accumulation of activated CD8 T-cells are the present in the brain [29], [47]. Results from the present study suggest that activated CD8 T-cells in the brain could foster an inflammatory milieu that inhibits neurogenesis, mediated in a large part by IFN-γ.

Several studies have shown that IFN-γ alters NSC properties; including inhibition of cell proliferation [32], [37], and driving differentiation into neurons or oligodendrocytes [41], [42], [48], [49], [50]. These effects are initiated by IFN-γ binding to its cognate receptor, which is widely expressed on NSCs and its derivative cells [37]. Data presented in the present study demonstrate that by blocking IFN-γ R1, either using a neutralizing antibody or IFN-γ R1 KO NSCs, effectively abrogates the inhibitory effects produced by activated CD8 lymphocytes, indicating that these effects are likely mediated through the IFN-γ receptor. The downstream signaling pathways initiated by IFN-γ binding that drives these changes in NSCs are poorly understood. NSCs treated with IFN-γ show increased Stat 1 phosphorylation [42], which in turn induces the expression of sonic hedgehog (Shh), a major brain morphogen [42], [50], [51]. These downstream signaling events are speculated to be involved in both dysregulation of NSC proliferation [50], [51] and the generation of a unique neuronal phenotype with irregular electrophysiological properties [42]. Data presented in our current study show that co-culture with activated CD8 T-cells downregulates nestin and Sox2 expression in NSCs, suggesting that activated CD8 T-cells may initiate a differentiation program in cultured NSCs through the IFN-γ signaling pathway. However, decreased Sox2 expression in NSCs directly correlated with decreased proliferation, a phenomenon that is not observed in cells that lack IFN-γ R1. This direct relationship between Sox2 expression with cell proliferation and differentiation has been shown in several cell types, including NSCs [52], [53], [54], [55]. In addition, our results demonstrate that activated CD8 T-cells may direct differentiation of NSC, driving them towards an altered astrocyte-like phenotype. Similar studies have shown that when induced to differentiate, IFN-γ drives NSC into a dysfunctional neuronal phenotype that express GFAP potentially skewing the reparative processes in response to brain injury [37].

Activated CD8 T-cells affect NSC function through different mechanisms. In the present study, we have demonstrated that antigen independent activation of CD8 T-cells suppresses NSC proliferation through an IFN-γ mediated mechanism. Wang et al (2010) report that soluble mediators like Granzyme B (GrB), produced by human CD8 T-cells activated by crosslinking CD3/CD28, inhibit NSC proliferation and drive differentiation of cells to an astrocyte phenotype [56]. This inhibitory effect of GrB is mediated through the induction of a voltage-dependent potassium channel, Kv1.3, on NSC. GrB signaling in NSC is sensitive to Pertussis toxin, indicating that it may be mediated through a G protein-coupled receptor [56]. It is noteworthy that the effects of T-cells on NSC function are dependent on both the inflammatory context and developmental stage at which these cells interact (reviewed in [57]). In fact, IFN-γ treatment of human embryonic stem cell derived neural progenitors, albeit their expression of IFN-γ R1, fosters different differentiation profiles compared to murine NSCs [58]. Similarly, T-cell derived IFN-γ production during acute HSV-1 brain infection is significantly higher than that expressed during the chronic phase [47]. Studies on the effect of various concentrations of IFN-γ have indicated that higher concentrations of IFN-γ may activate caspases and potentially induce NSC apoptosis [37]. Previous studies in our lab have shown that at low concentration, IFN-γ can inhibit NSC proliferating without inducing apoptosis [59]. In the present study, we show that at the ratios of T-cells tested, NSC proliferation is suppressed without the overt induction of apoptosis. In several brain diseases (like Multiple Sclerosis, and ischemic stroke) among humans, IFN-γ is present at relatively high concentrations (∼1000 pg/ml) in the cerebrospinal fluid [58] and understanding the interactions between soluble mediators produced by T-cells and is critical to developing therapeutic approaches, particularly when using stem cells.

In summary, the impact of CD8 T-cells on reparative processes in the inflamed brain is both complex and poorly understood. Our study demonstrates the antigen-independent effects of activated CD8 T-cells on NSCs is mediated by IFN-γ, through its effect on the IFN-γ R1 on NSCs. Antigen-dependent effects of CD8 T lymphocytes could be facilitated by IFN-γ-induced increases in MHC-1 expression on NSCs [32], [60] and may be unique to the antigens involved in the insult. Numerous immune mediators have been shown to modulate the reparative processes initiated at the NSC niches (reviewed in [61]), suggesting that understanding the inflammatory context is critical for evaluating the impact of immune cells on neurogenesis. The data presented in this study elucidate one of the many mechanisms that may be involved in immune modulation of adult neurogenesis.

## Supporting Information

Table S1 **IFN-γ Production by T Lymphocytes.**
(DOCX)
