# Supplementary material for: Activated CD8+ T Lymphocytes Inhibit Neural Stem/Progenitor Cell Proliferation: Role of Interferon-Gamma
Source: PLoS One. 2014 Aug 18;9(8):e105219. doi: 10.1371/journal.pone.0105219 (PMC4136865; doi:10.1371/journal.pone.0105219)
Supplement: Table S1 — IFN-γ Production by T Lymphocytes. (DOCX) [file pone.0105219.s001.docx]

**Table S1: IFN-γ Production by T Lymphocytes**

| Treatment | IFN-γ (pg/mL) | | |  |
| --- | --- | --- | --- | --- |
|  | **CD8 T cells** | | **CD4 T Cells** | |
|  | Unstimulated | Stimulated | Unstimulated | Stimulated |
| With NSCs | <10 | 30.4±3.9 | <10 | <10 |
| Without NSCs | <10 | 151.7±59.7 | <10 | <10 |
| ELISA limit of detection is 10pg/mL. Data presented are average (± SEM) of 5 separate experiments performed in triplicate. | | | | |
